# Menopause: a midlife crisis for women in India

**DOI:** 10.1136/bmjgh-2024-017863

**Published:** 2025-06-18

**Authors:** Nikita Haresh Rajput

**Affiliations:** 1 Division of Cancer Care, Hospital Cancer Registries and Survival Studies, Center for Cancer Epidemiology, Navi Mumbai, India

**Keywords:** Health policy, Aging, Public Health, India, Health education and promotion

**SUMMARY BOX**

- Menopause poses health risks in India, with rural women experiencing limited healthcare access, low awareness and higher rates of premature menopause.
- Healthcare differences between urban and rural areas might result in untreated symptoms and lack of awareness for menopausal women, negatively influencing their quality of life.Integrating alternative therapies like Ayurveda and Yoga into menopause care might offer culturally appropriate and holistic solutions.
- The study calls for action in government policies and to initiate focused health programmes for postreproductive women, which are crucial for improving healthcare for both urban and rural regions of India.

She says,

*“Ageing is a pause,”*

Sometimes with a cause,

But often seems like a loss,

and her life could go on a toss,

If we didn’t pause

To understand

Her menopause.

## Introduction

 Menopause is a natural biological transition for women, but in India, it poses unique challenges, especially for those in rural areas. Women not only experience physical changes but also shifts in the social behaviours of those around them. However, gaps in healthcare services, awareness and support systems continue to persist. In this context, implementing healthcare policies and fostering emotional support from family are crucial.

## Background

“Life expectancy is growing, while quality of life is still dormant.”

In India, more women are experiencing menopause due to increased life expectancy. The average age of natural and induced menopause is decreasing in urban and rural areas, respectively. The National Family Health Survey (NFHS-5) indicates that hysterectomies performed for fibroids or heavy bleeding can lead to early menopause.^1^ Factors, such as surgical menopause, low education, low income, rural living, female sterilisation, insomnia, depression and lack of insurance coverage, contribute to premature menopause.^2^,^4^ Many studies also overlook women who have had surgical menopause, potentially underestimating its prevalence. Early menopause can elevate risks for cardiovascular disease, urinary incontinence and sexual health issues.^5^ The transition to menopause begins years prior and includes stages, such as perimenopause, where women often experience symptoms like hot flushes, sweating and sadness. In contrast, postmenopausal women typically face joint and muscular soreness. Special attention is needed for perimenopausal women due to their heightened risk of physical and psychological disorders.

## Cultural perceptions of ageing and menopause

“Ageing is natural and requires our graceful acceptance.”

Because of societal stigmas connected with ageing, the majority of pharmaceutical corporations in developed nations promote hormone replacement therapy for symptom management. On the other hand, women in rural patriarchal contexts frequently lack basic information about assistance choices for symptom management.^6^ However, most studies on menopause research focus solely on women in metropolitan areas, leaving a gap in rural women’s experiences.

Menopausal responses differ greatly depending on genetics, culture, lifestyle, financial condition, education, behaviour and diet. Menopause is often viewed positively in rural India since it alleviates sociocultural constraints. Many rural women feel emancipated from the monthly constraints of menstruation, such as sanitary product costs, particularly in places with restricted water supply and also have social benefits, such as increased engagement in spiritual meetings and improved status. Women from lower socioeconomic backgrounds struggle for daily basic needs, which makes menopause symptoms less noticeable and frequently regards menopause as a natural aspect of ageing rather than a separate condition.^7^

In urban areas, managing somatic symptoms is more challenging, and in India, cultural norms hinder discussions about these issues with male family members, adding burden to their psychological health.

## Implementing policies and adopting alternative therapies

The earlier onset of menopause poses significant challenges for India’s healthcare system. While the government supports pregnant women and malnourished children, policies for menopausal women are lacking. Lifestyle modifications can help regulate hormonal levels and support the body’s natural progression towards menopause at the expected age.^8^ Although health initiatives like menstrual hygiene campaigns exist, alternative therapies for conditions, such as uterine fibroids and heavy bleeding, which could reduce the need for hysterectomies, are underused. Many doctors lack training in menopause and are unaware of effective alternative remedies. India has rich traditions of alternative therapies, such as Ayurveda and Yoga that should be implemented.

Recently, a letter to the Indian parliament (Lok Sabha) called for policies addressing menopause in both government and private sectors, prompting the Ministry of Women and Child Development to consider formulating such policies.^9^ However, the needs of rural women differ from those in urban women and may require greater awareness and medical support instead of leave policies.

## Call for policy changes and community support

To address these challenges effectively, the following framework outlines essential strategies for improving the quality of life for menopausal women in India (see table 1).

**Table 1:** Action plan for improving menopausal women’s health in India

*Component*	*Key actions*	*Expected outcomes*
*Resources*	- Train staff in menopause management - Diagnostic and prognostic tools	- Increased **capacity** for **care delivery**
*Interventions*	- Offer free health check-ups for women≥35 years of age - Implement awareness campaigns	- Enhanced **knowledge** of menopause
*Community engagement*	- Facilitate support groups and community events-Raise awareness through local leaders	- Increased **emotional support** and understanding
*Holistic care approach*	- Integrate diet, exercise and meditation into care plans	- Improved **quality of life** and well-being
*Policy support*	- Develop menopause policies in government and private sectors - Promote alternative therapies like Ayurveda and Yoga	- Improved **access** to healthcare services

Bold values focus on subject which needs to be payed attention, for expected outcomes of the health action plan.

The framework summarises the key components necessary for a comprehensive approach to menopause care, emphasising the need for holistic support, awareness campaigns and policy reforms.

## Conclusion

**Figure 1:**
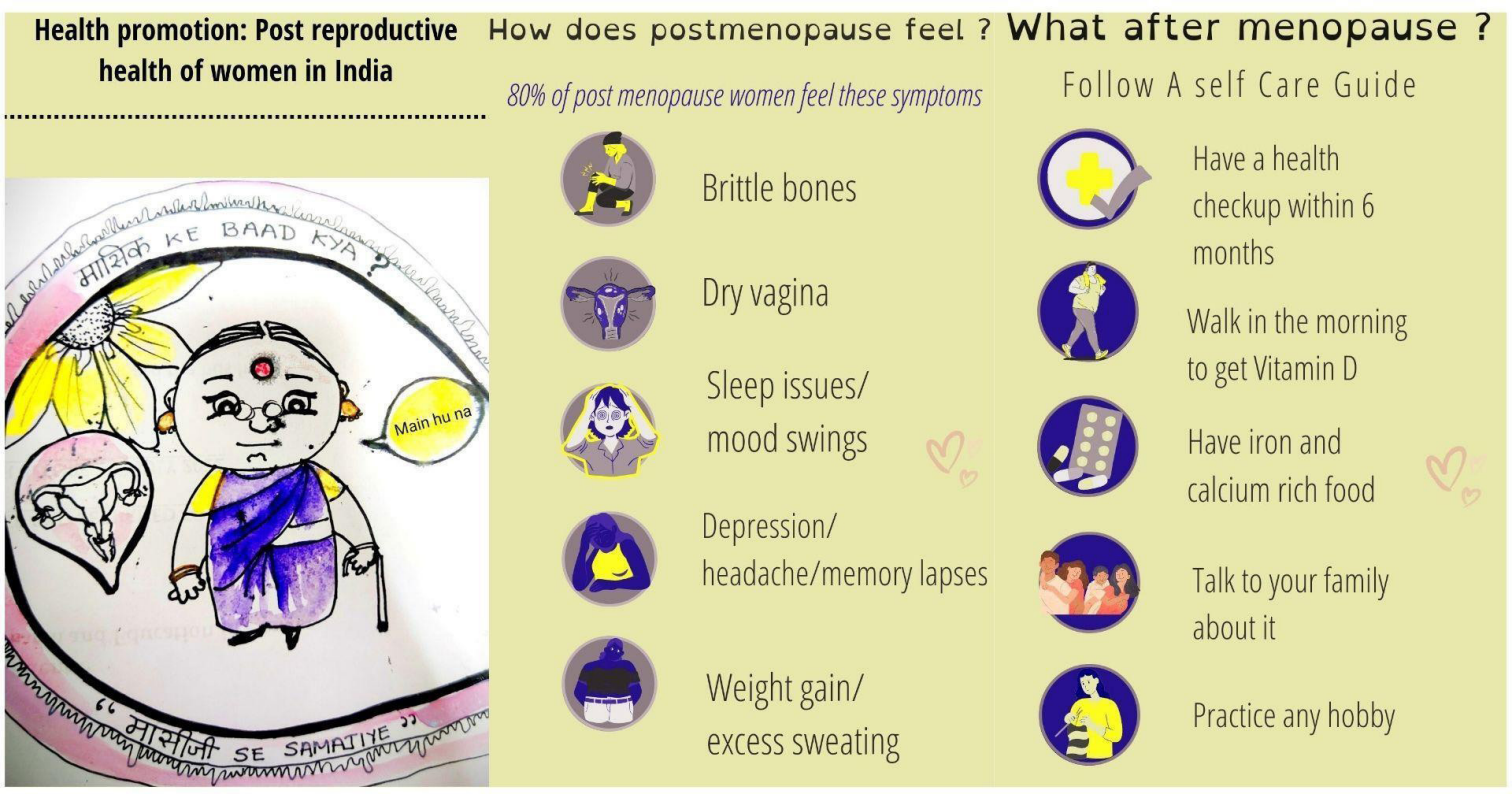
Health promotion strategies for postmenopausal women in India, illustrating common symptoms and recommended self-care practices. The figure shows an old female depicting a rural or urban region woman in India who is offering help to discuss menopause symptoms and a self-care guide for menopause. (Message in Hindi: ‘मासिक -Masik ke baad kya? –मासीजी -Massiji se samjiye’ – English Translation—‘What after Menopause?-Understand with aunt Massiji’ (general Hindi term used for mother’s sister).

Addressing the challenges of menopause in India requires a multifaceted approach that considers the unique needs of both rural and urban women. By implementing comprehensive policies and expanding healthcare services, we can create a supportive environment for all women navigating this critical life stage. As shown in figure 1, adopting self-care strategies, such as regular health check-ups, morning walks for vitamin D synthesis and a balanced diet rich in calcium and iron along with engaging in hobbies and maintaining open family communication, can significantly alleviate symptoms and enhance emotional well-being. Furthermore, equipping primary healthcare providers with the necessary training to offer holistic care, including lifestyle interventions like yoga and meditation, will greatly improve the quality of life for menopausal women. With dedicated effort and collaboration, we can foster a healthcare system that respects and addresses the diverse experiences of women, ultimately contributing to their overall well-being and empowerment.

The artwork presented in figure 1 is an original creation by the author and has not been copied, adapted or referenced from any external source, publication or website.
